# The Mitochondrial Carnitine Acyl-carnitine Carrier (SLC25A20): Molecular Mechanisms of Transport, Role in Redox Sensing and Interaction with Drugs

**DOI:** 10.3390/biom11040521

**Published:** 2021-03-31

**Authors:** Annamaria Tonazzi, Nicola Giangregorio, Lara Console, Ferdinando Palmieri, Cesare Indiveri

**Affiliations:** 1 Institute of Biomembranes, Bioenergetics and Molecular Biotechnologies (IBIOM), National Research Council, Via Orabona 4, 70126 Bari, Italy; a.tonazzi@ibiom.cnr.it (A.T.); n.giangregorio@ibiom.cnr.it (N.G.); 2 Unit of Biochemistry and Molecular Biotechnology, Department DiBEST (Biologia, Ecologia, Scienze della Terra), University of Calabria, Via P. Bucci 4C, 87036 Arcavacata di Rende, Italy; lara.console@unical.it; 3 Department of Biosciences, Biotechnologies and Biopharmaceutics, University of Bari, 70125 Bari, Italy

**Keywords:** carnitine, carnitine acyl-carnitine carrier, carnitine acyl-carnitine translocase, membrane transport, mitochondria, mitochondrial carrier, mitochondrial transporter, post-translational modification, solute carrier family 25, SLC25A20

## Abstract

The SLC25A20 transporter, also known as carnitine acyl-carnitine carrier (CAC), catalyzes the transport of short, medium and long carbon chain acyl-carnitines across the mitochondrial inner membrane in exchange for carnitine. The 30-year story of the protein responsible for this function started with its purification from rat liver mitochondria. Even though its 3D structure is not yet available, CAC is one of the most deeply characterized transport proteins of the inner mitochondrial membrane. Other than functional, kinetic and mechanistic data, post-translational modifications regulating the transport activity of CAC have been revealed. CAC interactions with drugs or xenobiotics relevant to human health and toxicology and the response of the carrier function to dietary compounds have been discovered. Exploiting combined approaches of site-directed mutagenesis with chemical targeting and bioinformatics, a large set of data on structure/function relationships have been obtained, giving novel information on the molecular mechanism of the transport catalyzed by this protein.

## 1. Introduction

**Figure 1:**
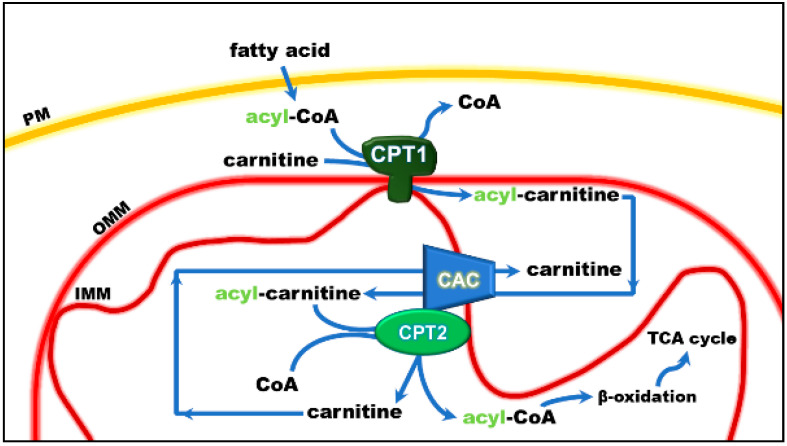
Role of the carnitine shuttle in the mitochondrial β-oxidation pathway. The shuttle is constituted by carnitine palmitoyltransferase 1 (CPT1) that converts acyl-CoAs into acyl-carnitines; carnitine/acyl-carnitine carrier (CAC) that allows the uptake of acyl-carnitines in the mitochondrial matrix in exchange with free carnitine, and carnitine palmitoyltransferase 2 (CPT2) that converts acyl-carnitines back to acyl-CoAs and releases free carnitine, which is ready to be translocated back to the cytosol by CAC. Once in the matrix, acyl-CoA undergoes β-oxidation with the production of acetyl-CoA that enters the tricarboxylic acid cycle (TCA). Other abbreviations: IMM, inner mitochondrial membrane; OMM, outer mitochondrial membrane; PM, plasma membrane.

The mitochondrial carnitine acyl-carnitine carrier (CAC) is the member A20 of the SLC25 protein family, including 53 solute transporters in humans [1,2,3], the majority of which are localized in the inner mitochondrial membrane. Until now, only one family member has been found in the peroxisomal membrane [4]. Furthermore, approximately one-third of them are still orphans, i.e., their transported substrates are unknown. This family members share a peculiar structural fold of six transmembrane segments characterized by 3-fold repeated couples of hydrophobic α-helices. Each couple is connected by a hydrophilic loop and contains the SLC25 sequence motif PX[D/E]XX[K/R] at about the boundary of the odd α-helix and the loop. The structural information on the SLC25 proteins derives mainly from the ADP/ATP carrier, which has been crystallized in both the outwards and inward open conformations [5,6]. All the other carrier structures have been predicted by homology modeling, including CAC, whose structure has been corroborated by site-directed mutagenesis and chemical targeting approaches. CAC is a key component of the carnitine shuttle [7], which is crucial for the mitochondrial β-oxidation pathway. In this shuttle (Figure 1), fatty acids are activated by the cytosolic acyl-CoA synthetase (ACSL) to fatty acyl-CoAs thioesters [8,9]. Since the mitochondrial inner membrane is not permeable to acyl-CoAs, acyl groups are transferred from CoA to carnitine by the action of “carnitine palmitoyltransferase-1a and b” (CPT-1a; CPT-1b), an integral outer membrane enzyme [10]. The acyl-carnitines cross the outer mitochondrial membrane through an almost unspecific pore constituted by the voltage-dependent anion channel (VDAC) [11] and, then, are specifically translocated across the inner mitochondrial membrane by the action of CAC. In the mitochondrial matrix, the enzyme carnitine palmitoyltransferase 2 (CPT-2) catalyzes the trans-esterification of the acyl groups from carnitine to mitochondrial CoA with the release of free carnitine, thereby providing acyl-CoA substrates for fatty acid β-oxidation. CAC and CPT-2 form a supramolecular complex in the inner mitochondrial membrane, devoted to acyl-carnitine channeling from the carrier to the enzyme (Figure 1) [12]. The carnitine released in this reaction is translocated backward to the cytosol by the same carrier via an acyl-carnitine/carnitine antiport reaction. The β-oxidation pathway is active in many tissues, especially those characterized by higher metabolic expenditure. It provides a large portion of the energy required by heart muscle, kidneys and also skeletal muscle, when glycogen has been consumed [13,14]. This pathway is also active in hepatocytes where fatty acid oxidation provides acetyl-CoA for ketone body synthesis during prolonged fasting conditions, in which glycogen stores have been depleted [15]. Neurons also perform fatty acid oxidation even though at a very low rate. Indeed, CAC also has been described in brain [16,17,18]. The crucial role of CAC in energy metabolism was demonstrated by the discovery of inherited defects of its gene *SLC25A20* causing secondary carnitine deficiency [19,20,21,22,23,24], a syndrome that arises in the very first stage of life as a life-threatening pathology. In this altered metabolic condition, acyl-carnitines fail to reach the mitochondrial matrix with consequent strong impairment of the β-oxidation. This syndrome is more severe than the primary carnitine deficiency caused by defects of the plasma membrane transporter OCTN2 (*SLC22A5)* [25,26,27]. Recent findings have correlated alterations of CAC expression or regulation with diabetes [28,29].

Unlike most mitochondrial carriers, which are obligatory antiporters [30], CAC can catalyze, besides the antiport reaction, also a unidirectional transport of substrates event though at a rate about one order of magnitude lower than the antiport [31,32]. Interestingly CAC is not only operating in animals but also yeast and plants. The *Saccharomyces cerevisiae* and the *Aspergillus nidulans* CACs share 29% and 42% identity with the human CAC, respectively [33,34,35,36]. The main function of these transporters, in contrast to that of mammalian CACs, is to transport acetylcarnitine rather than medium- and long-chain acyl-carnitines into mitochondria [33,37]. The plant CAC ortholog, identified based on the 37% sequence identity with the human counterpart, most probably plays a different role, that is, the transport of glutamate [38,39]. It is still not clear if CAC also operates in peroxisomes, where very long, branched-chain, and medium-chain fatty acids are imported [40,41].

The history of CAC started with the detection of an acyl-carnitine uptake into mitochondria, which was saturable, stereospecific, inhibitable, and temperature-dependent [42,43,44]. Then, the availability of methodologies capable of handling hydrophobic membrane proteins allowed us to purify the protein responsible for the observed transport phenomena. In 1990, a classical approach based on chromatography fractionation of a rat liver mitochondrial extract and on transport assay of the fractions by proteoliposome technology was adopted [45]. The purified protein was used for the first functional characterization [31,32,46,47,48]. Later, CAC was identified at a molecular level [19,49] and obtained on a large-scale by overexpression in *Escherichia coli* [50] by a procedure introduced in our laboratory for the bacterial overexpression of the oxoglutarate carrier [51] and recently named the expression, purification, reconstitution assay (EPRA) method [52]. The recombinant purified CAC was employed in studies of structure/function relationships, interaction with drugs and xenobiotics, and post-translational modifications that modulate its transport function [53,54,55,56,57,58,59,60,61,62,63,64].

This article, starting from some basic information on CAC, provides an up-to-date comprehensive overview of the most recent discoveries about its molecular mechanism of transport and the modulation/regulation of its transport function.

## 2. The Functional Role of CAC

**Figure 2:**
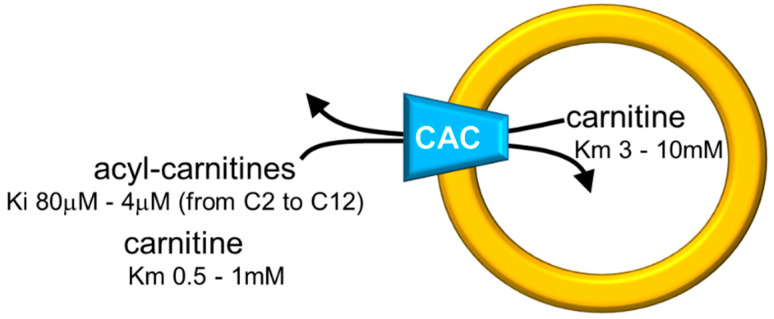
Sketch of the proteoliposome model with reconstituted CAC. Proteoliposomes contained 4% cardiolipin. Range of inhibition constants (Ki) for acyl-carnitines of various chain lengths on the external side and ranges of carnitine Km on both sides of CAC are reported.

Studies performed with intact mitochondria concurred to propose that the function of CAC in cells is that of catalyzing an antiport of acyl-carnitines with free carnitine according to the core activity of the carnitine shuttle [7] (Figure 1). Physiologically, the acyl-carnitines are transported from the cytosol to the mitochondrial matrix and the free carnitine in the opposite direction to sustain the intramitochondrial reactions of the β-oxidation pathway [42,65]. Later on, the studies in proteoliposomes confirmed this function. In the in vitro system, CAC purified from rat liver or recombinant CAC was inserted into the liposomal membrane with the same orientation as in the native membrane, thus representing a mitochondrion mimic single-protein model [32,50] (Figure 2).

The in vitro system allowed measurements of substrate affinity (Figure 2), giving further support to the preferential direction of transport of acyl-carnitines towards the internal space of proteoliposomes corresponding to the mitochondrial matrix and carnitine in the counter-direction. In vivo, this transport mode is driven by the higher acyl-carnitine concentration (see Figure 1) in the cytosol than in the matrix space, where acyl-carnitines are rapidly removed by the action of CPT2 that strictly interacts with the carrier [46,66]. Interestingly, the affinity profile of the carrier follows the specificity for acyl-carnitines of the CPT1 [67,68]. The possibility to manage the single-protein experimental system led to establishing that CAC catalyzes the transport of acetyl-carnitine too [54], suggesting that shorter carbon chain esters of carnitine across the inner mitochondrial membrane via CAC as the longer chain derivatives, in contrast to what previously hypothesized [69]. This indicates the role of CAC in participating in the scavenger action of acetyl-CoA from mitochondria [50,70,71]. In this frame, the matrix enzyme carnitine acetyltransferase converts acetyl-CoA to acetyl-carnitine, which can be exported from mitochondria in antiport with extramitochondrial carnitine. In this pathway, CAC works in a reverse mode mediating the efflux of carnitine derivatives from mitochondria [72]. However, a definitive demonstration of the reverse mode of action in vivo is still missing. The affinity of CAC for acetyl-carnitine is much lower than that for long-chain acyl-carnitines, at least on the external face of the carrier (Figure 2). Using the in vitro experimental system, a bisubstrate kinetic study carried out by varying both the internal and the external substrate concentrations demonstrated that CAC catalyzes the antiport of substrates according to a “ping-pong mechanism” [1,32,73]. This mechanism of transport involves only binary carrier substrate complexes and implies that CAC possesses a single “reorienting” binding site and two conformations, one with the substrate-binding site accessible from the cytosol and the other with the substrate-binding site accessible from the matrix. Therefore, the ping-pong mechanism, so named for the analogy with that of certain enzymes, is basically the same mechanism as the early hypothesized “single binding center-gating pore mechanism” [74,75] and as the recently described “alternating access mechanism” [5], which is based on numerous molecular details. It is worth mentioning that, according to the ping-pong mechanism, the Km for carnitine on the external or the internal side of CAC is influenced by the counter-substrate concentration, thus being variable within a certain range (Figure 2).

CAC also catalyzes a uniport reaction with a lower rate compared to the antiport. This almost unique feature among mitochondrial carriers was known since the 80s from studies in intact mitochondria [76] and later was confirmed by studies performed with proteoliposomes [31,32]. The rate of the unidirectional transport of carnitine is regulated by the counter-substrate; the uniport progressively decreases by increasing the concentration of the counter-substrate until the antiport mode is triggered. Physiologically, the net flux of carnitine allows for providing the matrix with carnitine newly synthesized in the cytosol or absorbed from the diet [31]. It must be stressed that (i) the last step of the carnitine biosynthesis occurs in the cytosol where the enzyme γ-butyrobetaine dioxygenase is located [77,78], (ii) the endogenous synthesis is not sufficient for the body’s needs, and (iii) more than 50% of carnitine is absorbed from the diet [79,80,81,82]. Therefore, the entire mitochondrial carnitine pool derives from extramitochondrial sources. As said before, CAC provides the matrix with carnitine through the uniport function. This role is crucial during mitochondrial biogenesis; however, no information is available on this issue. The uniport function should also be important for net export of carnitine to allow carnitine excretion and renewal. Very little information is available on the carnitine recycling. Indeed, even though it is known that an aliquot of carnitine is excreted through the urine, the flux of the molecule from the mitochondrial matrix to be excreted has never been dealt with.

Early studies showed the high sensitivity of CAC to sulfhydryl reagents [65,83]. Subsequent studies in proteoliposomes led to discrimination between two functional alterations caused by the reaction of SH reagents with two different cysteine populations: class-I cysteines are responsible for the induction of an “unphysiological” unspecific uniport; class-II cysteines are responsible for the inactivation of the carrier (both antiport and uniport function). On the one hand, the reaction of class-I cysteines with HgCl_2_ or mercurial derivatives, at relatively high concentration, converts the carrier to a “pore-like” transporter with reduced substrate specificity and uncoupling of the antiport function. This unphysiological activity reveals an intrinsic property of the mitochondrial carrier protein family members, i.e., a built-in channel normally hidden by appropriate gates [47,48] (and see Section 3). Indeed, this phenomenon also has been observed with other mitochondrial carrier proteins [84,85,86]. On the other hand, the reaction of class-II cysteines with HgCl_2_ and other mercurials at a low (nanomolar) concentration or with NEM and MTS leads to the inactivation of the transporter. Later, class-II Cys residues and the molecular basis of their inhibition were identified. This aspect will be dealt with in the following sections. In contrast, class-I Cys residues responsible for pore-like activity have not yet been identified.

Overexpression of recombinant CAC in *E. coli* [50] boosted the characterization of this transporter. Indeed, the recombinant protein showed the same properties as the native one indicating that it is suitable for functional studies. This breakthrough opened the perspective of studying the human CAC as well [34,87]. Novel functional information was achieved in a rather short time. The absolute need for cardiolipin, suggested by studies with the protein purified from rat liver, was clearly demonstrated with the recombinant CAC that is cardiolipin free and is inactive if not supplemented with the phospholipid [50,88,89]. Therefore, CAC belongs to the mitochondrial molecular systems, which require cardiolipin for an activity like many other mitochondrial carriers [4,90,91,92,93] or are modulated by cardiolipin as the NADH dehydrogenase [94,95]. Other important achievements following the involvement of recombinant CAC and site-directed mutagenesis strategy will be dealt with in the next section.

## 3. Structure-Function Relationships

The molecular basis of CAC substrate-binding and transport, as well as its regulation by post-translational modifications, have been explored using the site-directed mutagenesis approach complemented with bioinformatics and chemical targeting, together with parallel investigations in intact mitochondria to explore the physiological roles of these modifications. Together with those regarding the oxoglutarate carrier [96,97,98], the structure/function relationship studies concerning CAC are the most advanced within the mitochondrial carrier family members. These studies started with the construction of the homology model of CAC in its cytosolic open conformation based on the ADP/ATP carrier (AAC, *SLC25A4*) structure [99]. Later, the structural fold and dynamics have been updated with the homology model of the matrix open conformation of CAC obtained by using the recently solved AAC structure in its matrix open conformation as a template [5]. The molecular map of the amino acids involved in the catalytic process of CAC has been defined together with the role of specific residues in the molecular mechanism of transport and the regulation of the carrier function.

### 3.1. Substrate Binding Site and Translocation Events

**Figure 3:**
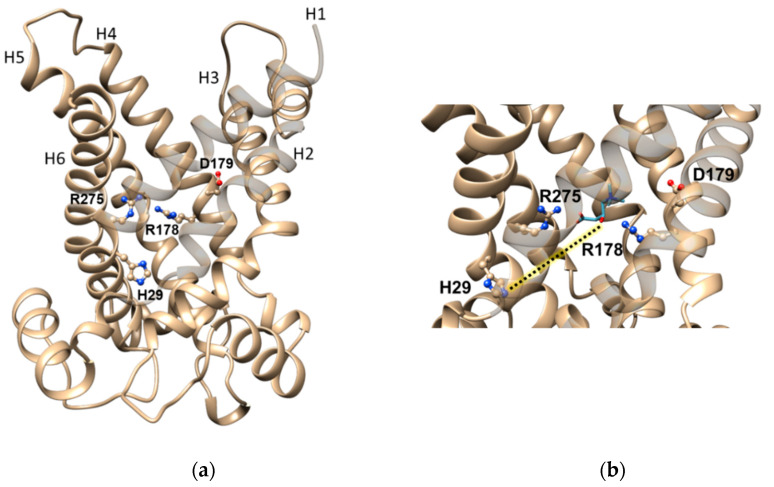
Ribbon diagrams of CAC showing the amino acids involved in carnitine binding. (**a**) Lateral view of the CAC structural model. The residues Arg-178, Asp-179, Arg-275, and His-29 are highlighted with a ball and stick representation. The transmembrane spanning α-helices are numbered. (**b**) Enlarged view of the residues interacting with carnitine. The dotted line indicates the following step in the translocation process in which carnitine will interact with His-29 before the matrix gate opens (see also Figure 5). Amino acid residues are dispayed with ball and stick rappresentation in which oxygen and nitrogen atoms are depicted in red and blu respectively. CAC model and carnitine position have been obtained as [87].

The residues responsible for substrate-binding were first hypothesized by bioinformatics in some yeast carriers, including the homolog of the human CAC [100,101]. Then, the identification of the amino acid residues of the mammalian CAC involved in substrate-binding and translocation has been conducted, exploiting site-directed mutagenesis, on the rat and human CACs that are virtually coincident, being 92% identical. The impairment or loss of function observed in conservative or non-conservative mutants, respectively, clearly demonstrated the role of each crucial residue in terms of the importance of the chemical features of the amino acid side-chains. As reported below, the amino acids involved in carnitine binding/translocation have been mapped. Asp-179, Arg-275, and Arg-178 (Figure 3a) undergo ionic and/or hydrogen bond interactions with carnitine, being involved in binding the trimethylammonium and the carboxyl groups, respectively. These charged residues that line the central water-filled cavity of the transport protein are conserved along with the CAC orthologs [87] according to their important role. The electric charges of the residues at positions 179 and 275 are more important than the side-chain length since the Vmax and/or the Km values show the greatest changes upon substituting the charged residues with neutral ones. In line with the crucial role of Arg-275, the point mutation Arg275Gln in the CAC of three patients was associated with severe carnitine deficiency [102]. His-29 is also conserved throughout the CAC sub-family members. The mutation of His-29 with Ala, Asp, Lys, Phe, Asn, or Tyr severely impairs the function. Only if His-29 is substituted by Gln, the activity of the transporter is comparable to that of the wild-type CAC. Indeed, the N amide of Gln structurally corresponds to the τ-N (distal) of the His-29 imidazole, indicating that the main role of His-29 in the formation of an H-bond with the substrate. This bond is established with the β-OH of carnitine or with the β-O- of acyl-carnitines. Therefore, His-29 plays a role in facilitating the correct positioning of the substrate preceding the translocation event towards the opposite side of the membrane (Figure 3b) [103].

**Figure 4:**
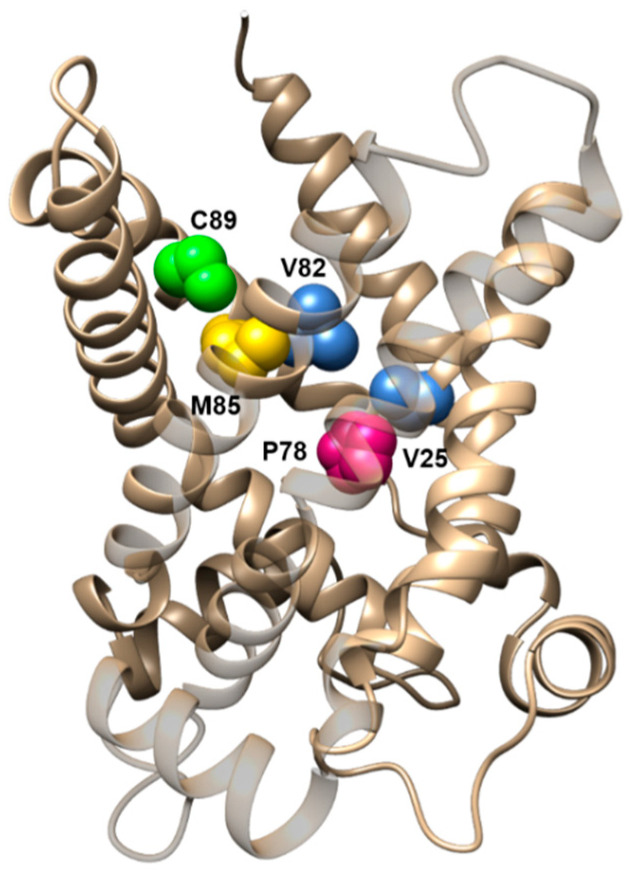
Lateral view of the CAC structural model highlighting the residues involved in binding the acyl moieties of acyl-carnitines. CAC residues are displayed with sphere representation.

Besides carnitine, acyl-carnitines are transported by CAC. These carnitine derivatives contain hydrophobic chains, esterified to the hydroxyl group of carnitine, with a length ranging from 2 (acetyl) to 16 (palmitoyl) or more carbon atoms. Val-25, Pro-78, Val-82, Met-84, and Cys-89, all belonging to the first and second transmembrane α-helices of the protein (H1-H2), constitute the “hydrophobic pocket” of CAC that binds the carbon chain of the acyl-carnitines (Figure 4). The ability of this “hydrophobic pocket” to interact with hydrophobic molecules correlates well with the higher average hydrophobicity of transmembrane α-helices H1 and H2 of CAC concerning that of the corresponding α-helices of the other members of the SLC25 family [66,100].

**Figure 5:**
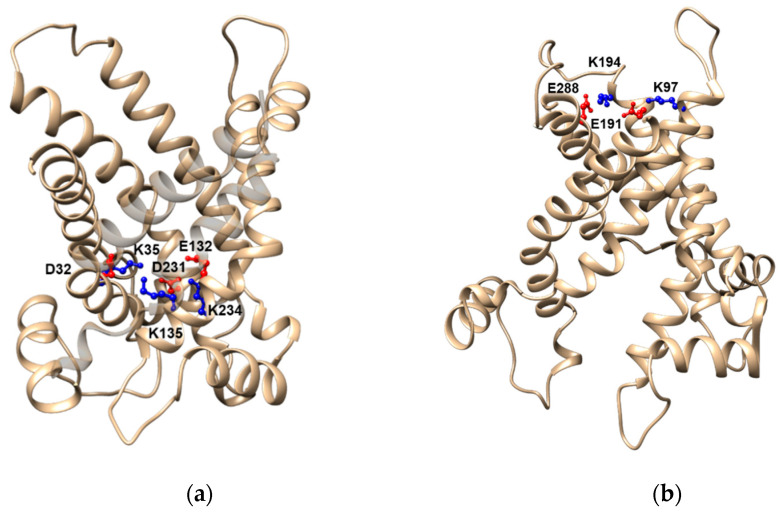
Lateral views of CAC highlighting the residues of the matrix and cytosolic gates. (**a**) The residues Asp-32, Lys-35, Glu-132, Lys-135, Asp-231, and Lys-234 forming the matrix gate are depicted in red (negatively charged residues) or in blue (positively charged residues); (**b**) the residues Lys-97, Glu-191, Lys-194, and Glu-288 forming the cytosolic gate are depicted in red (negatively charged residues) or in blue (positively charged residues).

Once the carnitine or the acyl-carnitine has interacted with the proper residues in the c-state, a charged gate constituted by the amino acid side chains of Asp-32, Lys-35, Glu-132, Lys-135, Asp-231, and Lys-234 (Figure 5a) located below the binding site, needs to be unlocked for the translocation to occur [99,104,105,106,107]. The six residues form three ion pairs resulting from the interactions between the couples: Asp-32 with Lys-135, Glu-132 with Lys-234, and Asp-231 with Lys-35. The role of these residues and their interactions have been validated using the mutagenesis approach [87]. Once the gate is opened by the fast interaction of the substrate with at least one of the charged residues, i.e., Lys-35, the carrier changes its conformation from the cytosolic state opened to the matrix opened state. The protein is stabilized in the matrix opened conformation by a gate, similar to the matrix one that is formed towards the cytosolic face and is composed of four charged residues, namely, Lys-97, Glu-191, Lys-194, and Glu-288 (Figure 5b). The other two residues of the cytosolic gate of CAC are uncharged in contrast to the corresponding residues of other carriers [96,106]. The free energy of the cytosolic gate of CAC is, therefore, lower than that of the matrix gate. This probably determines an imperfect coupling of the flux of substrates in the outward and inward directions, conferring to CAC the capacity to mediate a uniport reaction besides the antiport reaction [31,106].

### 3.2. The Molecular Basis of the Antiport Mode

**Figure 6:**
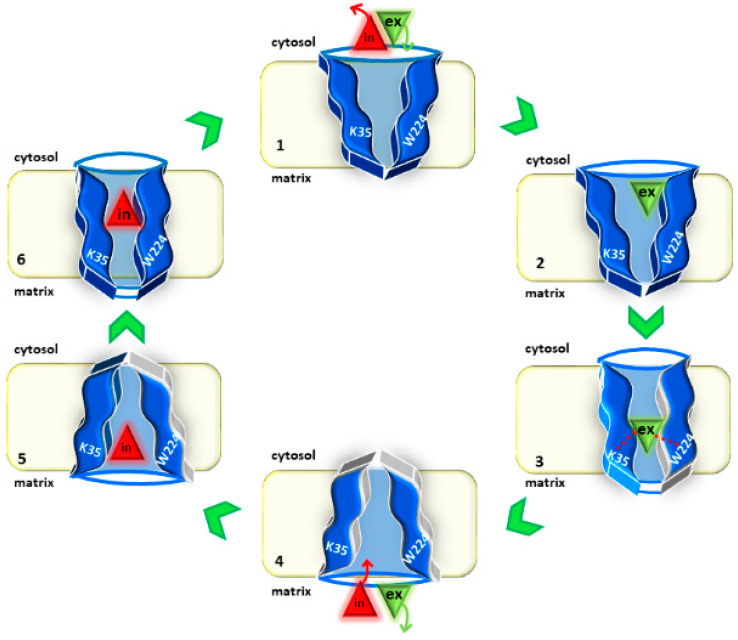
Sketch of the transport cycle of CAC. The states of the transporter during the antiport reaction are displayed: (1) c-state in the absence of substrate, (2) c-state with the external substrate entering the carrier, (3) occluded state or transition state with the external substrate bound to the substrate-binding site, (4) m-state in the absence of substrate, (5) m-state with the internal substrate entering the carrier, (6) occluded state or transition state with the internal substrate bound to the substrate-binding site. The role of K35 and W224 during the transport cycle is highlighted with dotted lines.

CAC shares with the other mitochondrial carriers the peculiar structure constituted by six transmembrane segments arranged in three intramembrane domains, which rotate to allow the conformational changes required for the transport reaction [5]. The antiport mode of transport is determined by the coupling of substrate-binding with gate opening on one side and gate closing on the other side. Indeed, given that the substrate considerably decreases the activation free-energy barrier of the carrier transition, the rate of transition of the unbound carrier from an outward open conformation (c-state) to the inward open one (m-state) or vice versa (Figure 6) is much lower than that of the substrate-bound carrier [108].

**Figure 7:**
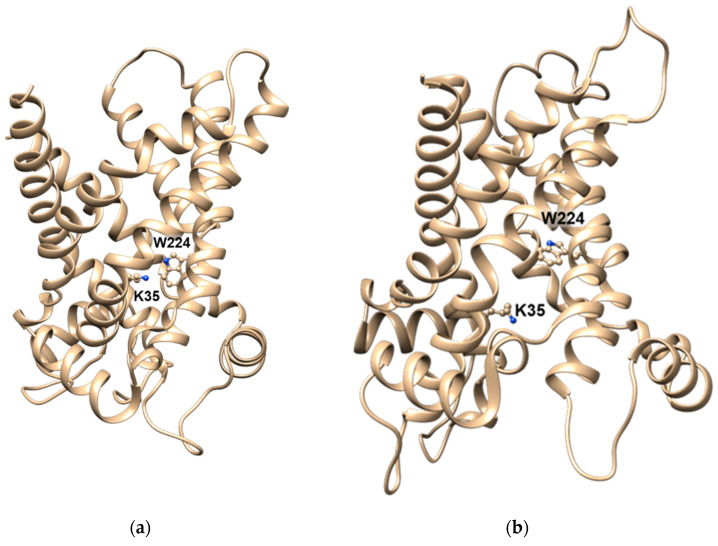
Lateral views of the CAC structural model showing the residues involved in the coupling of substrate-binding with gate opening and gate closing. (**a**) Ribbon diagram of the carrier in c-state in which the residues Trp 224 and K35 are at a distance of 4 Å and are depicted with a ball and stick; (**b**) ribbon diagram of the carrier in m-state in which the residues Trp 224 and K35 are at a distance of 12 Å and are depicted in ball and stick.

In the case of CAC, the identification of the residues, which are crucial for the coupling of substrate-binding with gate opening, was achieved by mutations that specifically abolish the antiport function without interfering with the uniport function. One of these residues is Lys-35, whose substitution with an uncharged residue impairs the antiport reaction, suggesting that Lys-35 interacts with the carboxyl group of carnitine favoring the gate opening [109]. The companion amino acid residue involved in binding the ammonium group of carnitine is Trp-224, whose substitution completely abolishes the antiport function and converts the protein into a uniporter with a specific activity and substrate specificity equal to those of the unidirectional transport activity of the wild-type CAC. The distance between Lys-35 and Trp-224 in the cytosolic open conformation (Figure 7a) corresponds to the distance between the ammonium and the carboxyl groups of carnitine, in line with the interaction of carnitine with these residues, which triggers the gate opening and closing. The distance between these residues increases in the matrix open conformation preceding the substrate release (Figure 7b). In the absence of the interaction of carnitine with Lys-35 and Trp-224, the CAC gate could open as well, but at a much lower rate constant leading to the uniport function. Trp-224 is conserved in all CAC sub-family members. Indeed, the substitution of this residue in the CAC of *A. nidulans* leads to the same alterations as in the mammalian transporter, indicating that the molecular determinant of the antiport function has been conserved during evolution [110]. Interestingly, a corresponding Trp residue is not present in the other proteins of the SLC25 family except in the ornithine/citrulline carrier, whose substrate, ornithine, harbors a positively charged amino group. In this carrier, the substitution of the Trp unveils a low rate of uniport activity [110,111].

## 4. CAC as a Redox Sensor

**Figure 8:**
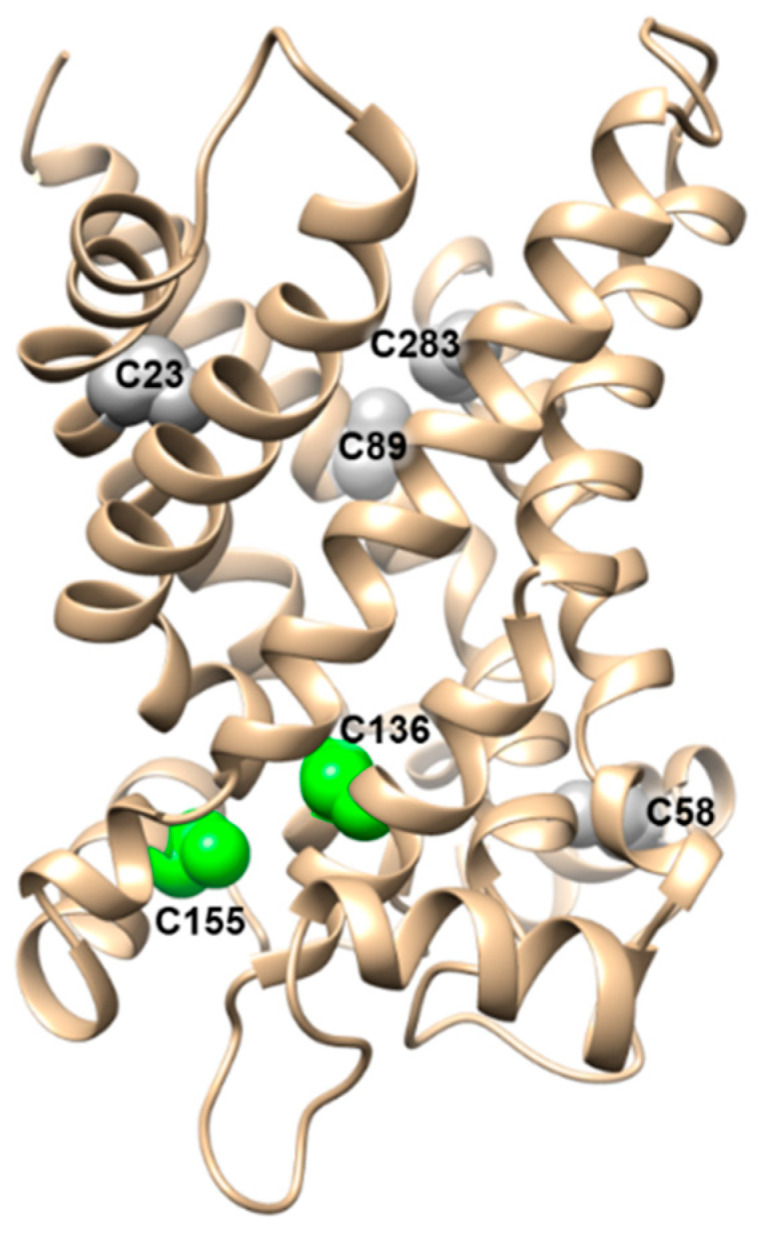
CAC structural model showing the two redox-sensitive cysteines. Lateral view of a ribbon diagram of the carrier in which the cysteine residues are displayed by a sphere representation. Cys-136 and Cys-155 are highlighted in green.

The capacity of CAC of interacting with thiol-reactive compounds was demonstrated initially in intact mitochondria [83], then confirmed and deepened in studies with the native protein by transport assays in proteoliposomes (see Section 3). However, the molecular determinants of the CAC redox sensitivity were identified only after the production of the recombinant CAC that gave the possibility to perform site-directed mutagenesis. Out of the six Cys residues of the protein, the sole Cys-136 and Cys-155 (Figure 8) are able to sense thiol-reagents at sub-micromolar concentrations as well as physiological effectors involved in cell redox sensing and control. Indeed, mutants in which one of the two Cys residues was substituted with Ser or Ala were less sensitive to thiol-reagents, and the mutant harboring the substitution of both Cys-136 and Cys-155 were mostly insensitive to reagents. Moreover, the mutant containing only Cys-136 and Cys-155, but lacking the other four Cys residues, exhibited the same reactivity as the wild-type protein. [88,112] The high sensitivity of the two residues is linked to their location in the core of the transport pathway, or in its vicinity, and to the local amino acid environment that confers peculiar properties to the cysteine thiol groups in terms of reactivity (pKa of the thiol groups) and propensity to undergo disulfide cross-linking. Indeed, some physiological or chemical reactants act on the “molecular sensor” constituted by Cys-136 and Cys-155. This cysteine couple, according to the oxidation state, behaves as an on–off switch of the protein. Indeed, if these two cysteines are oxidized to a disulfide, CAC is inactive due to a block of the conformational changes needed for the transition from the outward to the inward open conformation and vice versa. On the other way round, if the disulfide is converted to the thiol form of the Cys residues by a chemical or a physiological reactant, the CAC function is rescued [89]. Most likely, in vivo, the transporter exists as a mixture of the two states. Therefore, the actual transport capacity (specific activity) in vivo depends on the fraction of the protein, which is in the active (reduced) state. This is in line with the observation that the protein after extraction and isolation from the native membrane is not fully active. The maximal activity can only be observed after treating CAC with a strong reducing agent, such as DTE. The fraction of reduced (or oxidized) protein is variable and depends on incubation conditions of the mitochondria or on other factors, which cannot be precisely controlled in experiments. The redox sensing property of CAC allows its modulation by physiological effectors and, in turn, modulation of the β-oxidation pathway flux. The studies that will be resumed below uncover the molecular basis of the redox sensing feature of CAC.

### 4.1. Regulation of CAC by H_2_O_2_

CAC senses strong redox cellular changes through H_2_O_2_, an endogenous compound whose concentration can increase under oxidizing conditions, reaching millimolar levels locally, especially in mitochondria [113,114,115,116]. H_2_O_2_ inhibits CAC transport activity to an extent depending on both its concentration and time of reaction. H_2_O_2_ interacts with the thiol groups of Cys136 and Cys155, inducing the formation of a disulfide leading to inhibition. After shorter interaction times (0–2 min) with H_2_O_2_, sulfenic acid derivatives are formed, which evolve to disulfide (S-S) or to sulfinic (SO_2_^−^) and sulphonic (SO_3_^−^) species after longer reaction times (30 min). The reactions leading to sulfenic acid and disulfides can be reverted by reducing agents (glutathione), while those generating sulfinic and sulphonic acid are irreversible, thus abolishing the ability of the carrier to be switched-on by reducing agents [63]. Translating these data to cellular metabolism, it appears that low H_2_O_2_ levels regulate the activity of CAC, thus tuning the mitochondrial oxidation of fatty acids. At higher H_2_O_2_ concentrations, maintained for a longer time, which may occur under pathological conditions, the transporter is blocked, and fatty acid oxidation is arrested. Therefore, under conditions of strong oxidative stress, H_2_O_2_, acting on CAC as a signal molecule, may contribute to switching energy production from aerobic lipid metabolism to anaerobic glycolytic metabolism [117], causing a reduced oxygen consumption and a reduced formation of ROS. These effects can be interpreted as a protective mechanism against oxidative stress.

### 4.2. Regulation of CAC by Glutathione

Differently from oxidant species, such as H_2_O_2_ that “switch off” the CAC, reduced glutathione “switch on” the transporter acting on the disulfide between Cys-136 and Cys-155 [118]. Indeed, the physiological GSH/GSSG couple is, normally, present at a ratio of over 100/1, thus prevailing the reducing power. However, the ratio GSH/GSSG can change depending on the redox state of the cell. Thus, the CAC activity can also be modulated by the GSH/GSSG couple. Experimental data obtained by the EPRA method using the Cys mutants of CAC demonstrate that (i) GSH and GSSG cause activation or inhibition, respectively, acting on the SH/S-S exchange between Cys136 and Cys155 and (ii) the thiol group of Cys136 is the residue reacting first with GSSG. The action mechanism of these effectors implies the reversible glutathionylation of the transporter. In cells, the degree and the rate of conversion between the two forms may, in turn, depend on the activity of the enzyme Glutaredoxin-1 (Grx1) located in the intermembrane space of mitochondria [119,120] even though no direct evidence is provided so far of the contribution of this enzyme to the regulation of CAC by GSH/GSSG. CAC is the first mitochondrial carrier known to be responsive to the redox state of mitochondria and undergoes a full redox cycle from a reduced/activated state to an oxidized/inactivated state and vice versa. This responsiveness, again, relapses on the rate of fatty acid oxidation and hence on the production of ATP [118]

### 4.3. Modulation of CAC by NO

Nitrosylation processes modulate a huge number of cell pathways [121,122]. Row proteomic data indicated that CAC is targeted by NO among many other proteins [123]. When treating the native or the recombinant CAC with NO inhibitory effects can be observed [62]. The effects strictly depend on the presence of Cys-136, and the inhibition of transport is based on the steric hindrance caused by the NO-Cys bond close to the active site of CAC, where Cys-136 is located. CAC S-nitrosylation may occur under specific conditions in which mitochondrial oxidation of fatty acids must be slowed down, for example, to avoid CoA trapping by acetyl-CoA. Such conditions may intervene during impairment of the respiratory chain activity, which can be caused, among other motives, by increased intramitochondrial NO level and inhibition of complex I [124]. This phenomenon may control/regulate the fatty acyl flux into β-oxidation during altered mitochondrial metabolism, such as in ischemia and reperfusion. The NO-mediated inhibition of CAC may act in preventing the accumulation of reducing equivalents and decreasing ROS formation following re-oxygenation. Furthermore, the inhibition of CAC and the consequent impairment of β-oxidation may also contribute to the metabolic switch towards glycolytic metabolism [48,49] that has an important role in ischemic conditions [50].

### 4.4. Modulation of CAC by H_2_S

Beyond the previously described signals acting on CAC, it has been demonstrated that CAC is an H_2_S sensor. H_2_S is one of the endogenous gas transmitters (NO, CO and H_2_S), which is produced by several enzymatic pathways, two of them being mitochondrial [125,126,127,128,129]. H_2_S exerts its action on CAC interacting with the “crucial” Cys-136 and Cys-155 couple. Actually, it shows a higher affinity for Cys-155 compared to Cys-136, in contrast with H_2_O_2_, NO, and GSSG, which “prefer” Cys-136. This difference is related to the solubility, reactivity, and size of H_2_S (or HS^−^). H_2_S first reacts with Cys-155 forming –SSH; then the free -SH of Cys136 reacts with the –SSH producing the disulfide Cys136-S-S-Cys155, which inactivates CAC [89]. Furthermore, H_2_S in the solution can form polysulfides [130] which can reduce protein disulfides and, hence, convert the Cys-136/Cys-155 disulfide to free thiol groups. Such a reaction well explains the reactivation of the inhibited transporter observed after long time incubations. Thanks to its gaseous nature and its affinity for CAC, H_2_S manages the rate/flow of fatty acid mitochondrial β-oxidation, exerting a prompt and fine-tuned modulation of this pathway. Therefore, under certain conditions, e.g., those of oxidative stress, the H_2_S-mediated regulation of CAC activity can cause a switch of aerobic metabolism to glycolysis with consequent cardio-protection upon ischemia/reperfusion [61].

## 5. Other Regulatory Mechanisms

CAC is also a target of non-enzymatic acetylation processes, which lead to inhibition of its transport activity [60]. This mechanism, too, contributes to the regulation of the mitochondrial β-oxidation pathway. The effect of acetylation on CAC is opposite to that described for the citrate carrier (CIC, *SLC25A1*), which is activated by acetylation [131]. The different behavior of CAC and CIC versus acetylation correlates well with the roles of the two transporters in the β-oxidation and biosynthesis of fatty acids, respectively, because CAC is necessary for their β-oxidation and CIC is necessary for their biosynthesis. The acetylation of CAC exerts a dynamic control on the protein as the non-enzymatic process of acetylation can be followed by enzymatic deacetylation by the action of NAD^+^ dependent SIRT3. Notably, this process links the activity of CAC to the acetyl-CoA level in the mitochondria [60,132].

CAC is also subjected to transcriptional control. Its gene is located on chromosome 3p21.31, spans about 42 kb, and is split into nine exons with the translation start site in exon 1 [133,134]. This gene is differentially expressed in human tissues. High levels of transcripts are found in the liver, heart, and skeletal muscle, where β-oxidation is greatly exploited for energy production; much lower levels are observed in other tissues, such as the brain, placenta, pancreas, and lung [135]. Research on the proximal promoter has revealed the presence of binding sites for different transcription factors. For example, it has been demonstrated that an active binding site for PPARα is present in the CAC gene promoter at position −99/−80 bp and that PPARα is a strong activator of CAC gene expression [136]. CAC expression is also regulated by other transcription factors, such as PGC-1α and 1β, the estrogen-related receptor (ERR), the general factor Sp1, the specific factors FOXA2 and SRC-3, and possibly other factors not yet identified [137]. In addition, CAC gene expression is upregulated by drugs, such as statins, fibrates, and 9-cis-retinoic acid [138].

Another regulatory mechanism of CAC is exerted by the Micro-RNAs (miRNAs) 132 and 212, which lead to CAC suppression, causing inhibition of β-oxidation and accumulation of cellular long-chain fatty acyl-carnitine esters in pancreatic β-cells, ultimately leading to stimulation of insulin secretion. Interestingly, miRNAs 132 and 212 are upregulated in pancreatic β-cells in response to obesity in two mouse strains with different susceptibility to obesity-induced diabetes. Therefore, the downregulation of CAC may be a mechanism to enhance the insulin secretory response [28].

## 6. Interaction of CAC with Xenobiotics

An important aspect concerning the involvement of CAC in human health is its capacity to interact with xenobiotics. In addition, this ability is mostly related to the high reactivity of the Cys residues of the transporter. In some cases, the interaction occurs via the substrate-binding site or with a mixed mechanism. In the following sections, the activation or inhibition effects exerted by different xenobiotics are described and explained based on the chemical properties of each compound. Most of the interacting compounds are largely used or newly proposed drugs.

### 6.1. Polyphenols

The polyphenolic fraction extracted from several cherry cultivars is known for its antioxidant properties. Polyphenols can prevent CAC oxidation by atmospheric O_2_ or partially reverse protein oxidation by intracellularly-produced cell H_2_O_2_. The data obtained with the EPRA method highlighted that the antioxidant effect on CAC is mainly exerted by the compound 8 trans-3-O-feruloyl-quinic acid (3FQA), which is the most hydrosoluble/bioavailable and abundant in the cherry extracts. The last feature of this compound favors its approach to the substrate-binding site for reducing the disulfide between Cys136 and Cys155, which characterizes CAC under its oxidized state [139]. Therefore, the polyphenol action can improve mitochondrial functionality by acting on the fatty acid β-oxidation pathway. This, in turn, improves several defects correlated to elevated oxidative stress occurring in several diseases, such as Alzheimer’s disease, Down’s syndrome, and heart diseases.

### 6.2. Dantrolene

Dantrolene, a drug that possesses antioxidant properties [28] and is specifically used in the management of malignant hyperthermia, activates the oxidized (disulfide) fraction of CAC with a half-maximal effective concentration (EC50) of 9.3 μM. The effect of dantrolene on CAC activity also has been characterized with the EPRA method [58].

### 6.3. β-Lactam Antibiotics

The first report concerning the action of drugs on mitochondrial transport of carnitine dates back to 1994 [140]. The authors of this paper hypothesized that the toxicity of some β-lactam antibiotics was due to the inhibition of mitochondrial carnitine transport. However, it was only in 2008 when the molecular interaction of β-lactams with CAC was demonstrated [57] in liposomes reconstituted with the rat liver CAC. β-lactam antibiotics, which are among the most commonly used antibiotics in human therapy, are competitive inhibitors of CAC, probably due to their structural similarity with carnitine. In addition, they irreversibly bind CAC after longer incubation times and knock off carnitine transport [57]. In vivo, inactivation of CAC could impair fatty acid β-oxidation at a variable extent depending on the type of antibiotic and the therapy duration, leading to metabolic consequences of tissues, such as the liver or muscles, that greatly rely on fatty acid oxidation for energy production. Finally, the molecular interaction between β-lactam antibiotics and CAC, described above, may contribute to determining some mild side-effects of β-lactams [57].

### 6.4. Proton Pump Inhibitors

Omeprazole, a known K^+^/H^+^-ATPase inhibitor that is largely used to treat gastric acid-related disorders, also interacts with CAC. The molecular mechanism of interaction of omeprazole with CAC relies on forming S-S mixed disulfide(s) with Cys-136 or Cys-155, just as it does with the K^+^/H^+^-ATPase. Interestingly, omeprazole interacts with Cys-136, as other previously characterized sulfhydryl reagents, but also with Cys-283, which is not targeted by other reagents of the same type. The reaction with both Cys residues leads to the complete inactivation of the transporter. According to computational analysis, two omeprazole molecules are involved in this interaction, one for each Cys residue. In vivo, these implications deserve attention for their impact on the process of fatty acid β-oxidation leading to a mild carnitine deficiency-like syndrome [56,141].

### 6.5. Mildronate

Mildronate, an anti-ischemic drug also used as performance-enhancing, is a competitive inhibitor of CAC. It not only interacts with the substrate-binding site of CAC, but it is also transported by this carrier due to the high similarity with carnitine. The administered mildronate is taken up by the cells via OCTN2 and then inhibits acyl-carnitine transport into the mitochondrial matrix. Moreover, the matrix taken up mildronate acts on intramitochondrial metabolism enzymes. Therefore, CAC has a crucial role in the molecular mechanisms underlying the effects of mildronate [142].

### 6.6. Ingenols

Among several protein targets of ingenols, a class of drugs used for actinic keratosis, there is CAC. The carrier is inhibited by ingenol mebutate (IngMeb), which thus blocks acyl-carnitine uptake into the mitochondria and, hence, the mitochondrial fatty acid oxidation. The discovery that IngMeb and its more stable analog ingenol disoxate (IngDsx) inhibit CAC contributes to explain, at the molecular level, some of the mitochondrial defects observed in cells treated with high concentrations of these drugs [55].

### 6.7. Heavy Metals

CAC plays a major role in mercury toxicology, being one of the most crucial targets of mercury. Indeed, the mercury compounds mercury chloride and methylmercury inactivate CAC in vitro and in vivo at submicromolar concentrations [59], which are at concentrations lower than those, which inactivate thioredoxin [143]. Using zebrafish as an animal model and HeLa cells as a human cell model, it has been demonstrated that mercury impairs the viability of zebrafish and human cells, respectively, at concentrations corresponding to those present in the environment after pollution. By parallel experiments performed using the EPRA method, the molecular mechanism of action has been disclosed. The compounds act by targeting the transporter via mercury-thiol bonds with Cys-136 and Cys-155. The data correlate well with the previous findings on CAC purified from rat liver [47]. From a physiological point of view, this is relevant since the average concentration of mercury in human tissues (about 0.15 μM) can increase to more than 5 μM upon acute or chronic exposure to pollutants [144]. Overexposure to mercury causes mitochondrial toxicity by the chemical CAC knocking off [59]. As mercury, also copper exerts a strong inhibition on CAC, even though at higher concentrations [64]. The effect and the mechanism of interaction between copper and CAC have been defined by site-directed mutagenesis and computational chemistry approaches. The oxidation state of the cation does not influence its effectiveness as an inhibitor since Cu^2+^ and Cu^+^ show the same IC50. The mechanism of interaction with CAC consists of the formation of a cross-link among the copper ion and the two Cys-136 and Cys-155 residues. This cross-link, similarly to the disulfide between the two Cys residues, “switches off” the transporter [64]

## 7. Conclusions

The carnitine acyl-carnitine carrier (CAC) has a long history being one of those membrane transporters whose study started with its functional characterization in intact mitochondria and continued with its biochemical description and investigation at the molecular level using the native purified protein or the bacterially expressed recombinant purified protein. The more recent studies of CAC, performed by joining up-to-date methodological approaches, such as in vitro transport assay, site-directed mutagenesis, and bioinformatics, confirmed and extended previous findings as well as discovered molecular mechanisms of its transport activation and inhibition. Thus, several regulatory properties of CAC, which are based on post-translational modifications of Cys or Lys residues of the transporter, emerged from these studies. In particular, two specific Cys residues behave like an on–off switch of the carrier, responding to signals of physiological effectors, such as GSH, hydrogen sulfide, and nitric oxide. Altogether, the summarized studies highlight the transporter’s involvement in fatty acid metabolism, suggesting a central role of CAC in controlling the β-oxidation pathway in response to the redox state of the cell. Therefore, CAC represents an exciting drug target.

## Abbreviations

CAC: carnitine acyl-carnitine carrier; EPRA: expression, purification and reconstitution assay; NEM: N-ethylmaleimide; MTS: methanethiosulfonate reagents; IngMeb: ingenol mebutate; IngDsx: ingenol disoxate; SLC25: solute carrier family 25.
